# Overexpression of transport proteins improves the production of 5-aminovalerate from l-lysine in *Escherichia coli*

**DOI:** 10.1038/srep30884

**Published:** 2016-08-11

**Authors:** Zhong Li, Jing Xu, Tongtong Jiang, Yongsheng Ge, Pan Liu, Manman Zhang, Zhiguo Su, Chao Gao, Cuiqing Ma, Ping Xu

**Affiliations:** 1 State Key Laboratory of Microbial Technology, Shandong University, Jinan 250100, People’s Republic of China; 2 School of Life Sciences & Biotechnology, Shanghai Jiao Tong University, Shanghai 200240, People’s Republic of China

## Abstract

Bacterial transporters mediate the exchanges between intracellular and extracellular environments. Modification of transport route could be applied to speed up the metabolic reactions and promote the production of aimed compounds. Herein, lysine 2-monooxygenase (DavB) and δ-aminovaleramidase (DavA) were co-expressed in *Escherichia coli* BL21(DE3) to produce nylon-5 monomer 5-aminovalerate from l-lysine. Then, PP2911 (4-aminobutyrate transporter in *Pseudomonas putida*) and LysP (the lysine specific permease in *E. coli*) were overexpressed to promote 5-aminovalerate production using whole cells of recombinant *E. coli*. The constructed *E. coli* strain overexpressing transport proteins exhibited good 5-aminovalerate production performance and might serve as a promising biocatalyst for 5-aminovalerate production from l-lysine. This strategy not only shows an efficient process for the production of nylon monomers but also might be used in production of other chemicals.

Polyamides (also known as nylons), owing to their excellent material properties in mechanical strength, melting point, corrosion resistance, and other superior qualities, have been applied extensively in industrial and medical fields^1^,^2^,^3^. The basic materials for polyamides production are generally acquired from petrochemicals. As concerns about environmental issues and petroleum resource availability continue to grow, it is critical that we produce viable alternatives from cost-effective renewable resources. Nylon-4,6 has been commercialized and outpaces nylon-6 and nylon-6,6 in melting and thermal stability^4^. Nylon-5, which is produced from the homopolymerization of 5-amniovalerate, exhibits similar general properties to nylon-4,6^4^. What is significant is that 5-amniovalerate could be produced through biotechnological routes, which guarantees the green route to nylon-5 production. 5-Aminovalerate could also serve as a potential C5 platform chemical to produce 5-hydroxyvalerate, glutarate, and 1,5-pentanediol^5^. In this regard, it is of high importance to develop methods for bio-synthesis of 5-aminovalerate.

5-Aminovalerate is an intermediate of l-lysine catabolism that is called aminovalerate pathway in *Pseudomonas putida*^6^. The transformation process is composed of two steps: oxidation of l-lysine into 5-aminovaleramide catalyzed by lysine 2-monooxygenase (DavB) and hydrolysis of 5-aminovaleramide into 5-aminovalerate catalyzed by δ-aminovaleramidase (DavA). Great efforts have been made for fermentative production of l-lysine^7^,^8^,^9^,^10^,^11^,^12^,^13^,^14^,^15^,^16^,^17^. The annual production of l-lysine is estimated at a large quantity of 2,200,000 tones and falling prices will result from the overcapacity of l-lysine synthesis^8^. In this context, it would be desirable and economically feasible to produce 5-aminovalerate using l-lysine as a starting material.

Significant researches have been devoted to explore methods for 5-aminovalerate production, for example, enzymatic synthesis^18^,^19^ and whole cell biotransformation^20^. It is universally believed that whole cells are more stable catalyst compared to isolated enzymes due to the protected environment provided by the cell membranes. However, the cell membranes could also be a main barrier for the reactions between substrates and the catalytic enzymes^21^. Meanwhile, the production process is limited by the accumulation of final products in the cytoplasm^22^. One general and efficient solution to these problems is to overexpress transport proteins. Bacterial transport proteins are kinds of transmembrane binding proteins of physiological importance in selective permeation of certain molecular. The metabolic process is accelerated with enhancement of substrate import and transportation of final metabolites from cytoplasmic enzymes. In this study, we constructed a novel recombinant *Escherichia coli* strain by overexpressing transport proteins (Fig. 1), which exhibited good 5-aminovalerate production performance.

## Results

### Feasibility of 5-aminovalerate production by recombinant *E. coli*

Firstly, the genes *davA* (gene ID: 1044092) and *davB* (gene ID: 1044093) of *P. putida* were inserted into the MCS1 of pETDuet-1 (Fig. S1A) to construct pETDuet-DavAB (Fig. S1B) which was then transferred into *E. coli* BL21(DE3). The 5-aminovalerate production performance of the resulting recombinant strain *E. coli* pDAB was verified accompany with *E. coli* pD harboring blank vector pETDuet-1 and *E. coli* pDB harboring recombinant plasmid pETDuet-DavB (Fig. S1D). The details for genetic manipulation and catalytic reaction process are presented in “Methods” section.

**Figure 2:**
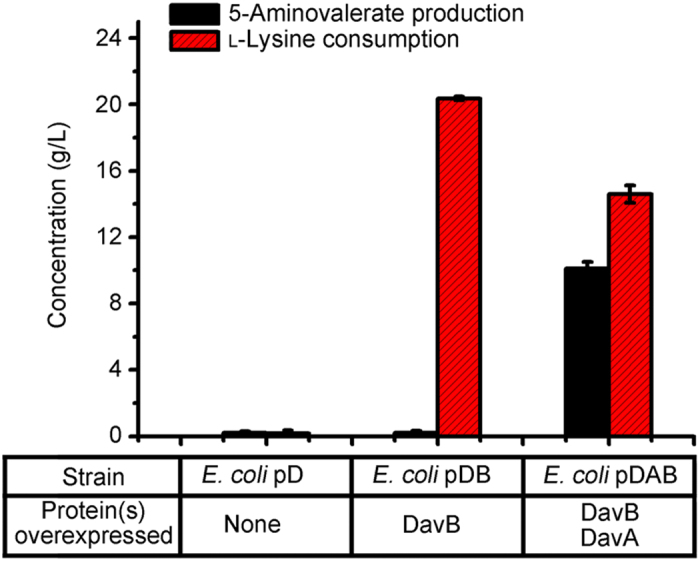
Verification of 5-aminovalerate production by different recombinant *E. coli* strains. Results are means ± SD of three parallel replicates.

As shown in Fig. 2, *E. coli* pD was unable to assimilate l-lysine and no 5-aminovalerate was accumulated. The inability of *E. coli* pD to consume l-lysine implied the suitability of *E. coli* as a host for efficient l-lysine conversion. *E. coli* pDAB was capable of converting 14.6 g/L l-lysine into 10.1 g/L 5-aminovalerate in 24 h, at a yield of 0.86 mol/mol. Although l-lysine was consumed, no 5-aminovalerate was detected during the whole reaction process with *E. coli* pDB. These results suggested that both DavB and DavA should work efficiently in recombinant *E. coli* for the 5-aminovalerate production. On the other hand, the *E. coli* pDB exhibited a higher l-lysine consumption rate than that of *E. coli* pDAB (Fig. S2A), implying the intracellular 5-aminovalerate might constrain the conversion of l-lysine.

### Effect of transport proteins on 5-aminovalerate production

To elevate 5-aminovalerate production, an improvement of l-lysine absorption was proposed to serve as an efficient approach. LysP is the specific lysine permease in *E. coli* with high affinity to l-lysine (apparent *K*_*m*_ of 2.5 μM)^23^. The gene *lysP* (gene ID: 946667) in *E. coli* K12 was amplified and integrated into the MCS2 of pETDuet-DavAB (Fig. S1C) to construct strain *E. coli* pDABL. Protein PP2911 of *P. putida* KT2440 was annotated as 4-aminobutyrate transporter. 4-Aminobutyrate transporters from *Arabidopsis thaliana*^24^ and mammals^25^ were capable of transporting 5-aminovalerate in addition to 4-aminobutyrate. In order to increase the export rate of 5-aminovalerate, the gene *pp2911* (gene ID: 1042844) from *P. putida* KT2440 was inserted into pACYCDuet-1 (Fig. S1E) to construct pACYCDuet-PP2911 (Fig. S1F). It was then transferred into *E. coli* pDAB to construct *E. coli* pDABP. The 5-aminovalerate production performance of *E. coli* pDABL and *E. coli* pDABP was then examined.

As shown in Table 1, the recombinant strain *E. coli* pDABP overexpressing DavB, DavA and PP2911 enabled to convert 22.4 g/L of l-lysine to 16.2 g/L 5-aminovalerate in 24 h. The introduction of PP2911 into *E. coli* pDAB leads to a production improvement by 60.4% and a yield increase to 0.90 mol/mol. However, *E. coli* pDABL yielded 6.9 g/L 5-aminovalerate from 11.5 g/L l-lysine in 24 h, which failed to show advantage compared with *E. coli* pDAB (Fig. S2). According to the results, the export of 5-aminovalerate rather than the absorption of l-lysine acted as the major factor limiting 5-aminovalrate production.

In order to explore whether the co-expression of LysP and PP2911 has positive effect on 5-aminovalerate production, the plasmid pACYCDuet-PP2911 was transferred into *E. coli* pDABL to construct *E. coli* pDABLP. As expected, the recombinant strain *E. coli* pDABLP successfully produced 16.9 g/L 5-aminovalerate from 22.5 g/L l-lysine in 24 h with a yield of 0.94 mol/mol (Table 1). Among all the constructed strains, the recombinant strain *E. coli* pDABLP overexpressing transport proteins LysP and PP2911 attained the best 5-aminovalerate production performance in titer, productivity and yield.

### Optimization of biocatalysis conditions of *E. coli* pDABLP

To elevate 5-aminovalerate productivity of *E. coli* pDABLP, the biocatalysis conditions were optimized. The effect of reaction temperature on production rate was determined. The influence of l-lysine concentration on the yield of 5-aminovalerate was examined. The conversion rate and catalytic activity which was defined as the 5-aminovalerate produced by per unit biocatalyst per hour were determined to optimize the biocatalyst concentrations. The specific optimization process is addressed in “Methods” section.

**Figure 3:**
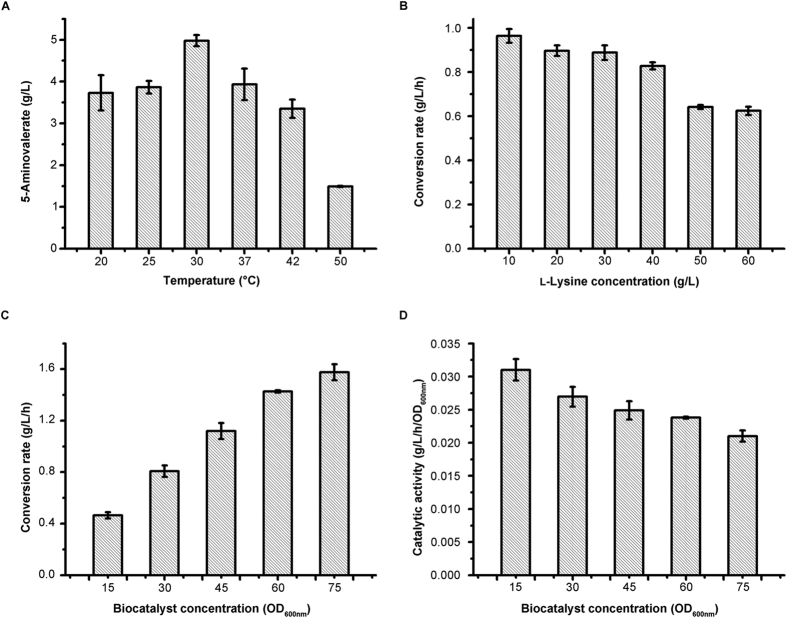
Optimization of biocatalysis conditions using strain *E. coli* pDABLP. (**A**) Temperature. (**B**) l-Lysine concentration. (**C**,**D**) Biocatalyst concentration. Results are means ± SD of three parallel replicates.

As shown in Fig. 3A, the optimal temperature was determined to be 30 °C. The 5-aminovalerate concentration increased as reaction temperature increased up to 30 °C and decreased thereafter. The yield of 5-aminovalerate maintained over 0.80 mol/mol when the l-lysine concentration below 40 g/L, and decreased sharply with higher l-lysine concentration (Fig. 3B). Thus, 40 g/L of l-lysine were chosen for further study. As expected, the conversion rate increased with the increase of biocatalyst concentration (Fig. 3C), while the catalytic activity decreased (Fig. 3D). Taken all associated factors into consideration, an OD_600nm_ of 60 was used for further research. Finally, the optimum catalytic conditions were determined to be reaction temperature, 30 °C; substrate concentration, 40 g/L; and biocatalyst concentration, OD_600nm_ = 60.

### Production of 5-aminovalerate under optimal conditions

**Figure 4:**
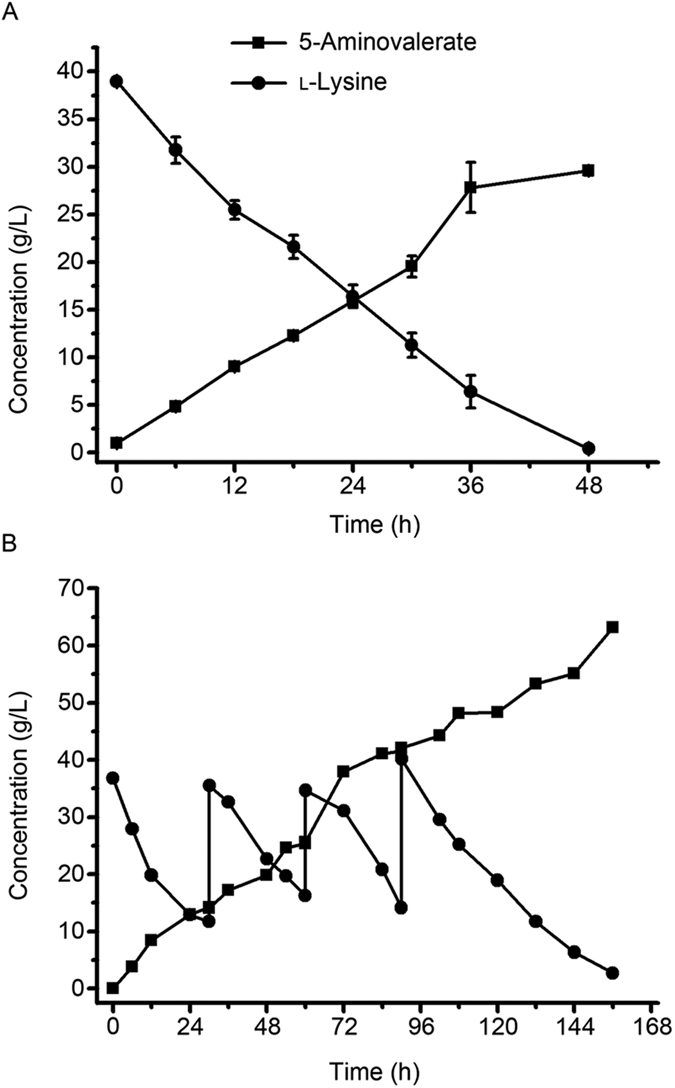
Batch (**A**) and fed-batch (**B**) process of 5-aminovalerate production from l-lysine by the recombinant strain *E. coli* pDABLP under optimized conditions. The reactions were carried out in a 300-ml conical flask containing 50 ml reaction mixture: 1/15 M PBS (pH 7.0), l-lysine and cell suspension (OD_600nm_ = 60). The reaction broth was incubated in a water baths shaker at 30 °C with shaking at 120 rpm. For the batch biotransformation process, results are means ± SD of three parallel replicates.

According to the results above, the bioconversion of l-lysine into 5-aminovalerate was conducted under optimized conditions. As shown in Fig. 4A, 29.6 g/L 5-aminovalerate was produced from 38.6 g/L l-lysine in 48 h. The yield of 5-aminovalerate was 0.96 mol/mol. Then the fed-batch process was performed to evaluate the production capacity of *E. coli* pDABLP. The time-course of 5-aminovalerate production from l-lysine was shown in Fig. 4B. The 5-aminovalerate concentration increased continuously in 156 h to a final titer of 63.2 g/L. In total, 102.3 g/L l-lysine was consumed. The yield of 5-aminovalerate from l-lysine was 0.77 mol/mol. The lower yield compared with batch biotransformation process might be caused by the instability of DavA, as mentioned in the previous research^18^.

## Discussion

Bio-based and biodegradable polymers have drawn worldwide attention as a result of environmental concerns and stress of limitation of petroleum resources. Polylactides stands at the forefront of practical use. Besides lactate, the biomonomers used to form polymers have been widely extended to succinic acid, 1,4-butanediol, 1,3-propanediol, terephthalic acid *et al*.^26^,^27^. 5-Aminovalerate, the precursor of nylon-5, has attained much attention recently^5^,^18^,^19^,^20^,^28^. l-Lysine, the original material of 5-aminovalerate, is generally manufactured by direct microbial fermentation of molasses or starch. Since the overcapacity of l-lysine synthesis has resulted in its falling price, the biotransformation of l-lysine, such as 5-aminovalerate production, might extend the scope of application and be beneficial to l-lysine industry.

Membrane-associated transporter proteins play an irreplaceable role in signal transduction, osmotic regulation and substance transportation. Metabolic engineering of transport route has long been applied to speed up the reaction and promote the production of aimed compounds. The lysine specific permease, LysP, is able to accelerate l-lysine uptake by 10- to 20-fold^29^. The expression of LysP was regulated by extracellular concentration of lysine and was shut off when l-lysine concentration was higher than 25 μM^30^. In a previously study, overexpression of LysP in *E. coli* BL21(DE3) caused the improvement of l-pipecolic acid production by 5-fold^31^. In this context, it is beneficial for LysP expression in l-lysine based biotransformation process. 4-Aminobutyrate transporters from different kingdoms showed high affinity for 5-aminovalerate in addition to 4-aminobutyric acid. In this work, LysP was overexpressed for lysine import while PP2911 was overexpressed for 5-aminovalerate export. PP2911 alone caused 5-aminovalerate production improvement by 60.4% and a yield increased to 0.90 mol/mol. Co-expression of LysP and PP2911 further improved the 5-aminovalerate production performance of the recombinant strain.

Several biotechnological routes have been used to produce 5-aminovalerate from l-lysine. Enzymatic synthesis of 5-aminovalerate from l-lysine was achieved by using lysine α-oxidase from *Trichoderma viride*^19^ or by coupling DavA and DavB from *P. putida*^18^. The maximum production of 5-aminovalerate catalysed by purified enzymes was 20.8 g/L^18^. Owing to the complexity of implementation and high operating cost, enzymatic production of 5-aminovalerate is still impractical in industrial field. Park *et al*. converted 7 g/L l-lysine into 3.6 g/L 5-aminovalerate through co-expression of *davAB* gene of *P. putida* in *E. coli* W3110^5^. Considering the low yield of 5-aminovalerate, they developed a new fed-batch fermentation process through which the mixture of l-lysine, glucose and MgSO_4_ was gradually added to reactor. At last, 5-aminovalerate was accumulated to a titer of 90.6 g/L^20^. In the present study, a new recombinant strain *E. coli* pDABLP based on *E. coli* BL21(DE3) was constructed to produce 5-aminovalerate from l-lysine. By overexpressing transport proteins LysP and PP2911, the result recombinant strain was able to transform more l-lysine than the strain only expressing DavA and DavB during batch biotransformation and the yield of 5-aminovalerate increased from 0.86 to 0.94 mol/mol. Although 63.2 g/L 5-aminovalerate was produced through a fed-batch transformation process, a higher concentration of 5-aminovalerate is expected to be achieved once the fermentative system developed by Park *et al*. is introduced.

5-Aminovaleramide, the sole intermediate of 5-aminovalerate production, belongs to primary amides with potential application value. The rapid l-lysine consumption rate of *E. coli* pDB implied the feasibility of 5-aminovaleramide production while *E. coli* lacks the enzyme to transform 5-aminovalerate as far as we know. The content of 5-aminovaleramide was not quantified because of the lack of commercially available 5-aminovaleramide. In the fed-batch biotransformation process (Fig. 4B), the continually l-lysine consumption in 156 h suggested the stability of DavB. In this regard, the recombinant *E. coli* pDB could serve for the efficient production of 5-aminovaleramide from l-lysine.

Production of C3, C4 platform chemicals such as glycerol, lactate, 3-hydroxypropinate, succinate, 2,3-butanediol, 1,4-butanediol and butanediamine^32^,^33^ has been extensively studied in recent years. However, only a few researches have focused on C5 platform chemicals production^34^,^35^. l-Lysine might serve as a suitable raw material for production of C5 difunctional alkanes. The l-lysine decarboxylase catalyzed the decarboxylation of l-lysine to produce cadaverine which could transform into 5-aminovaleraldehyde through subsequent transamination^36^. 5-Aminovaleraldehyde could be oxidized by 4-aminobutyraldehyde dehydrogenase to produce 5-aminovalerate^37^. Then, 5-aminovalerate could convert into glutarate by using δ-aminovalerate aminotransferase (DavT) and glutaric semialdehyde dehydrogenase (DavD)^6^. Glutarate semialdehyde, the only intermediate metabolite of glutarate production, could also be reduced into 5-hydroxyvalerate by NADH at the presence of 5-hydroxyvalerate dehydrogenase^38^. Accordingly, it is desirable and feasible to explore methods for biochemical production of C5 platform chemicals from directed biotransformation of l-lysine. In this study, the transformation of l-lysine into 5-aminovalerate was realized and accelerated with the co-expression of transport proteins. Further conversion of l-lysine into glutarate via 5-aminovalerate is under way based on the achieving results.

## Conclusion

We constructed a series of recombinant *E. coli* strains based on the aminovalerate pathway in *P. putida* to produce 5-aminovalerate from l-lysine. Acceleration of substrate import and product export was carried out by overexpressing transport proteins LysP and PP2911. The final constructed strain *E. coli* pDABLP exhibited good 5-aminovalerate production performance and might serve as an important biocatalyst for 5-aminovalerate production from renewable biomass.

## Methods

### Materials

l-Lysine, 5-aminovalerate and isopropyl-β-d-1-thiogalactopyranoside (IPTG) were purchased from Sigma-Aldrich (St. Louis, MO, USA). Polymerase chain reaction (PCR) primers were obtained from Sangon (Shanghai, China). Ampicillin and chloramphenicol were purchased from Amresco (USA). Restriction enzymes were obtained from Fermentas (Lithuania). T4 DNA ligase and fastPfu DNA polymerase were purchased from MBI (USA) and Transgen Biotech (China), respectively. All other chemicals were of analytical grade and commercially available.

### Strains, plasmids, genetic methods and culture conditions

*E. coli* DH5α was used as the host strain for gene manipulation. *E. coli* BL21(DE3) was used for protein expression. The genes coding DavAB, DavB, and PP2911 were amplified by PCR from genomic DNA of *P. putida* KT2440 using primers DavAB.f/DavAB.r, DavAB.f/DavB.r and PP2911.f/PP2911.r (Table 2) respectively. The *lysP* gene was cloned from *E. coli* K12 chromosomal DNA with primers LysP.f/LysP.r (Table 2). The resulting DNA fragments were digested with corresponding restriction endonuclease and inserted into expression vector pETDuet-1 (Fig. S1A) or pACYCDuet-1 (Fig. S1E).

All of the *E. coli* strains were cultivated in Luria-Bertani (LB) medium at 37 °C and 180 rpm. When necessary, ampicillin (100 μg/ml) and chloramphenicol (40 μg/ml) were added.

### Biotransformation by recombinant *E. coli* strains

For whole cell biocatalyst preparation, the recombinant *E. coli* strains harboring different plasmids were cultivated to the early exponential phase (OD_600nm_ = 0.4–0.6) at 37 °C. Then, 1 mM IPTG was added to the culture medium to induce protein expression. The culture was incubated at optimized temperature of 25 °C for additional 12 h. Cells were harvested by centrifugation (6000 rpm, 10 min), washed twice with 1/15 M phosphate buffer solution (PBS, pH 7.0).

To verify the 5-aminovalerate production by recombinant *E. coli* strains and the effect of transport proteins on 5-aminovalerate production, reactions were carried out in a 50-ml conical flask containing 10 ml reaction mixture: 1/15 M PBS (pH 7.0), about 20 g/L l-lysine and cell suspension (OD_600nm_ = 30). The reaction broth was incubated in a water baths shaker at 30 °C with shaking at 120 rpm. Samples (0.2 ml) were withdrawn at timed intervals and centrifuged (12,000 rpm, 15 min) before analysis.

### Optimization of biocatalysis conditions

The effects of catalytic conditions on biotransformation were determined in a 50-ml flask containing 10 ml reaction mixture: 1/15 M PBS (pH 7.0), l-lysine and cell suspension at 120 rpm. Only one parameter changed once while the others remained constant. To determine the effect of temperature on 5-aminovalerate production, the initial concentration of l-lysine was set as approximately 20 g/L. The OD_600nm_ of the cell suspension was set as 30. Samples were taken after incubation of 3 h at various temperatures (20, 25, 30, 37, 42, 50 °C). The effect of l-lysine concentration on the yield of 5-aminovalerate was determined at 30 °C and the OD_600nm_ was set as 30. Samples were taken after incubation of 3 h with different l-lysine concentrations (10, 20, 30, 40, 50, 60 g/L). To determine the effect of the whole cell concentration, the reactions were conducted with approximately 40 g/L l-lysine at 30 °C. Samples were taken after incubation of 12 h with various concentrations of whole cells (OD_600nm_ = 15, 30, 45, 60, 75). The samples (0.2 ml) were centrifuged (12,000 rpm, 15min) before derivatization and analyzation.

### Analytical methods

Cell density was measured at 600 nm (OD_600nm_) using an Ultrospec^™^ 2100 pro UV/visible spectrophotometer. Cells were diluted to suitable multiples in 1/15 M PBS (pH 7.0) to ensure the accurate measurement. 5-Aminovalerate and l-lysine concentration were quantified using a high-performance liquid chromatography (HPLC) system (Agilent 1100 series, Hewlett-Packard) equipped with a UV-Vis detector^18^. The samples were diluted to suitable multiples, and 400 μl of diluted samples was derivatized with phenylisothiocyanate (PITC)-acetonitrile (100 mM, 200 μl) and triethylamine-acetonitrile (1 M, 200 μl) for 1 h at room temperature before being extracted by *n*-hexane (800 μl). The lower aqueous phase was injected, separated on an Eclipse XDB-C18 (5 μm, 4.6 mm × 150 mm) maintained at 38 °C^39^ and detected at 254 nm. The mobile phase (at a flow rate of 0.6 ml/min) was composed of a solution of (A) pH 6.5, 100 mM ammonium acetate-acetonitrile (97:3) and (B) acetonitrile. The percentage of A declined from 82% to 70% in the first 20 minutes and maintained at 70% in the following 20 minutes.

## Additional Information

**How to cite this article**: Li, Z. *et al*. Overexpression of transport proteins improves the production of 5-aminovalerate from l-lysine in *Escherichia coli. Sci. Rep.* **6**, 30884; doi: 10.1038/srep30884 (2016).

## Supplementary Material

Supplementary Information

## Figures and Tables

**Figure 1:**
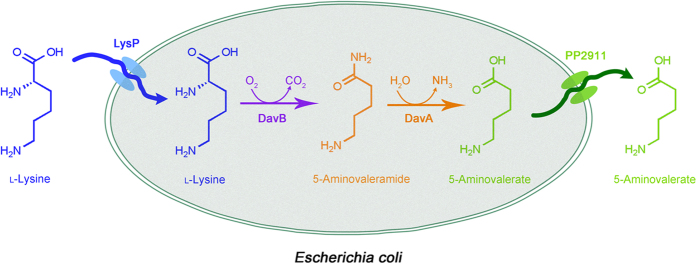
Scheme for the production of 5-aminovalerate from l-lysine by engineered *E. coli*. DavB, lysine 2-monooxygenase; DavA, δ-aminovaleramidase; LysP, lysine specific permease; PP2911, 4-aminobutyrate transporter.

**Table 1:** Overview of 5-aminovalerate production by recombinant *E. coli* strains^a^.

Strain	Proteins overexpressed	Titer^b^ (g/L)	Productivity^c^ (g/L/h)	Yield^d^ (mol/mol)
*E. coli* pDAB	DavA, DavB	10.1 ± 0.4	0.42 ± 0.02	0.86
*E. coli* pDABL	DavA, DavB, LysP	6.9 ± 0.4	0.29 ± 0.02	0.75
*E. coli* pDABP	DavA, DavB, PP2911	16.2 ± 0.2	0.68 ± 0.01	0.90
*E. coli* pDABLP	DavA, DavB, LysP, PP2911	16.9 ± 0.2	0.70 ± 0.01	0.94

^*a*^Data are the means ± SD of three parallel replicates.

^*b*^The final concentration of 5-aminovalerate.

^*c*^The rate of 5-aminovalerate production in recombinant *E. coli* strains.

^*d*^The mol of 5-aminovalerate produced per mol of l-lysine.

**Table 2:** Strains, plasmids and primers used in this work.

Strains, plasmids and primers	Structure or description	Source
Strains		
* E. coli* DH5α	*supE44* Δ*lacU169 (φ80 lacZΔM15) hsdR17 recA1 endA1 gyrA96 thi-1 relA1*	Novagen
* E. coli* BL21(DE3)	*F*^-^ *ompT hsdSB (rB*^*−*^*mB*^*−*^*) gal (λ c I 857 ind1 Sam7 nin5 lacUV5 T7gene1) dcm (DE3)*	Novagen
* E. coli* pD	*E. coli* BL21(DE3) harboring vector pETDuet-1	This study
* E. coli* pDB	*E. coli* BL21(DE3) expressing DavB	This study
* E. coli* pDAB	*E. coli* BL21(DE3) expressing DavAB	This study
* E. coli* pDABL	*E. coli* BL21(DE3) expressing DavAB and LysP	This study
* E. coli* pDABP	*E. coli* BL21(DE3) expressing DavAB and PP2911	This study
* E. coli* pDABLP	*E. coli* BL21(DE3) expressing DavAB, LysP and PP2911	This study
Plasmids		
pETDuet-1	Expression vector, ampicillin^r^	Novagen
pETDuet-DavB	N-terminal His-tagged *davB* gene in pETDuet-1	This study
pETDuet-DavAB	N-terminal His-tagged *davAB* gene in pETDuet-1	This study
pETDuet-DavAB-LysP	Both *davAB* and *lysP* with His-tag in pETDuet-1	This study
pACYCDuet-1	Expression vector, chloramphenicol^r^	Novagen
pACYCDuet-PP2911	N-terminal His-tagged *pp2911* gene in pACYCDuet-1	This study
Primers	Sequence (5′→3′)	
DavAB.f	GGATCCGATGAACAAGAAGAACCGCCAC (BamHI)	This study
DavB.r	AAGCTTTCAATCCGCCAGGGCGAT (HindIII)	This study
DavAB.r	AAGCTTTCAGCCTTTACGCAGGTG (HindIII)	This study
LysP.f	CATATGATGGTTTCCGAAACTAAAAC (NdeI)	This study
LysP.r	GGTACCTTATTTCTTATCGTTCTGCGG (KpnI)	This study
PP2911.f	GGATCCGATGCAAACCCACAAGAACAAT (BamHI)	This study
PP2911.r	AAGCTTTCAGGCGCCCTGCCCTA (HindIII)	This study
