# Supplementary material for: Overexpression of transport proteins improves the production of 5-aminovalerate from l-lysine in Escherichia coli
Source: Sci Rep. 2016 Aug 11;6:30884. doi: 10.1038/srep30884 (PMC4980613; doi:10.1038/srep30884)
Supplement: Supplementary Information [file srep30884-s1.pdf]

Supplementary Information

Overexpression of transport proteins improves the production of 5-aminovalerate from L-lysine in *Escherichia coli*

Zhong Li[a], Jing Xu[a], Tongtong Jiang[a], Yongsheng Ge[a], Pan Liu[a], Manman Zhang[a], Zhiguo Su[a], Chao Gao[a,]*, Cuiqing Ma[a] and Ping Xu[b]

[a] State Key Laboratory of Microbial Technology, Shandong University, Jinan 250100, People's Republic of China. E-mail: jieerbu@sdu.edu.cn; Tel: +86-531-88369463; Fax: +86-531-88369463

[b] School of Life Sciences & Biotechnology, Shanghai Jiao Tong University, Shanghai 200240, People's Republic of China.

*Corresponding author

Authors' emails:

ZL: lizhong302@sina.com

JX: 18769785368@163.com

TJ: jiangtt1797@126.com

YG: geyongsheng123@foxmail.com

PL: 15863136845@163.com

MZ: zmmsdu@163.com

ZS: sdsuzhiguo@qq.com

CG: jieerbu@sdu.edu.cn

CM: macq@sdu.edu.cn

PX: pingxu@sjtu.edu.cn

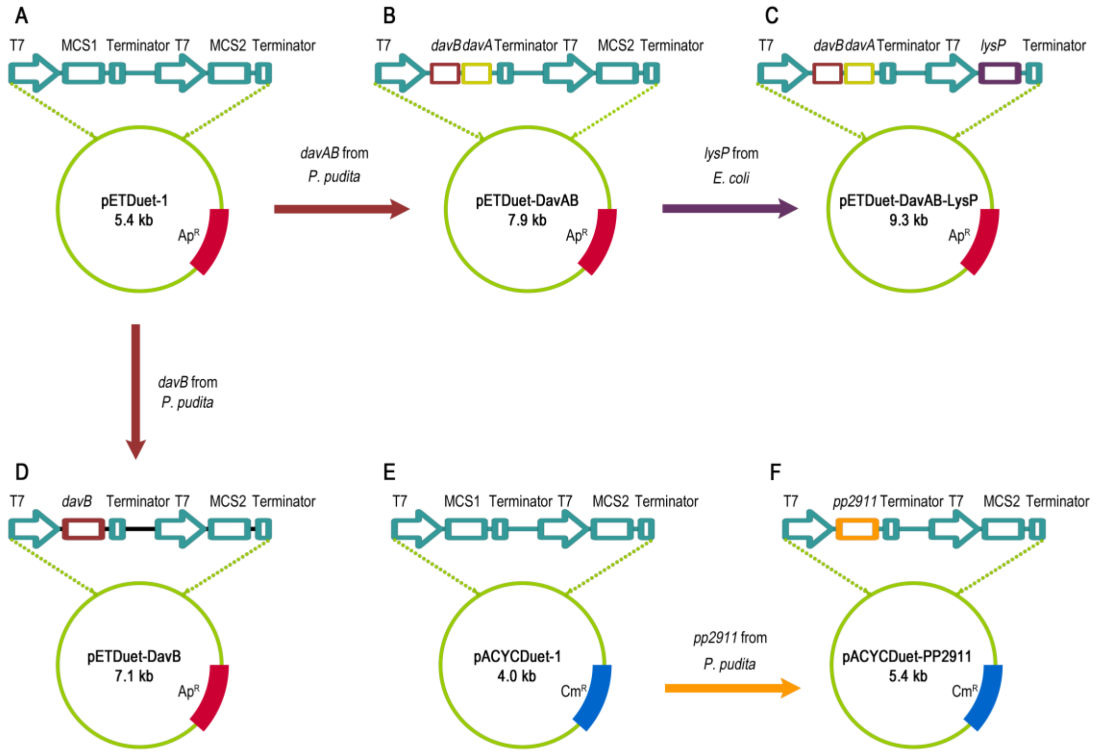

Fig. S1 Schematic representation of plasmids construction. A, pETDuet-1; B, pETDuet-DavAB; C, pETDuet-DavAB-LysP; D, pETDuet-DavB; E, pACYCDuet-1; F, pACYCDuet-PP2911.

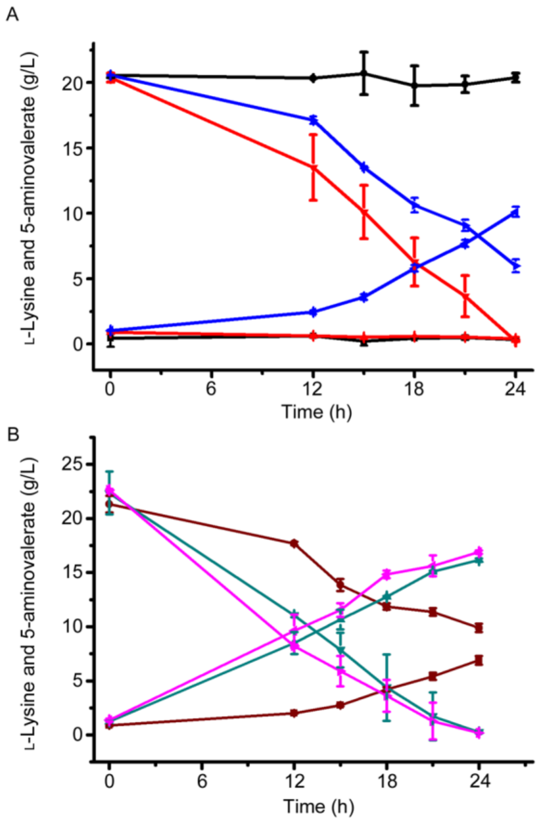

Fig. S2 Time-course of L-lysine consumption and 5-aminovalerate accumulation by different recombinant *Escherichia coli* strains. A, *E. coli* pD (●/■), *E. coli* pDB (▼/▲) and *E. coli* pDAB (▶/◀); B, *E. coli* pDABL (●/■), *E. coli* pDABP (▼/▲) and *E. coli* pDABLP (▶/◀). Reactions were carried out in a 50-ml conical flask containing 10 ml reaction mixture: 1/15 M PBS (pH 7.0), about 20 g/L L-lysine and cell suspension (OD_{600nm} = 30). The reaction broth was incubated in a water baths shaker at 30 ℃ with shaking at 120 rpm. Results are means ± SD of three parallel replicates.
